# Applying Trial Emulation Methods to Population-Scale Linked Electronic Health Records for Individuals with Multiple Long-Term Conditions Whilst Adjusting for Informative Observations.

**DOI:** 10.23889/ijpds.v9i5.2884

**Published:** 2024-09-18

**Authors:** Keith Abrams, James Rafferty, Jane Lyons, Ronan Lyons, Rhiannon Owen

**Affiliations:** 1 University of Warwick; 2 Swansea University

## Background & Objectives

Trial Emulation methods can be applied to Population-Scale Linked Electronic Health Records (EHR) to generalise the results of Randomised Controlled Trials (RCTs) to a broader patient population. However, the Data Generating Mechanisms (DGMs) associated with EHR data mean that there is a need to adjust for informative observations, i.e. worse prognosis patients may have more observations.

## Approach

Using the Anglo-Scandinavian Cardiac Outcomes Trial-Blood Pressure Lowering Arm (ASCOT-BPLA) as a case study: a RCT of a calcium-channel blocker-based regimen versus a β-blocker-based regimen, an emulated trial was created from population-scale, individual-level, linked anonymized EHR data curated as part of the Wales Multimorbidity e-cohort (WMC) for individuals who would have been eligible for ASCOT-BPLA but who also had Multiple Long-Term Conditions (MLTCs). Outcomes were systolic and diastolic blood pressure estimated/predicted at treatment initiation, 6 weeks, 3 months, and 6 monthly until 4 years.

## ResuMethods & Resultslts

Predictions were made using a fully probabilistic Bayesian joint model which allowed for dropout due to death and which either did or did not also adjust for the frequency of blood pressure observations available. In previous simulation studies such a joint modelling approach has been shown to out-perform other adjustment methods in terms of bias and coverage.

## Conclusions & Implications

Whilst EHR data offer an attractive platform in which to evaluate interventions in people living with MLTCs, the inherent DGMs associated with them need to be taken into account, and the use of a joint modelling approach is an attractive way to achieve this.

